# Association between Neck Circumference and Chronic Kidney Disease in Korean Adults in the 2019–2021 Korea National Health and Nutrition Examination Survey

**DOI:** 10.3390/nu15245039

**Published:** 2023-12-08

**Authors:** Youngmin Yoon, Yoo-min Kim, Somin Lee, Byung-Chul Shin, Hyun-Lee Kim, Jong-Hoon Chung, Minkook Son

**Affiliations:** 1 Division of Nephrology, Department of Medicine, Chosun University Hospital, Chosun University School of Medicine, Gwangju 61453, Republic of Korea; korean8503@chosun.ac.kr (Y.Y.); dlthals0425@naver.com (S.L.); bcshin@chosun.ac.kr (B.-C.S.); hyunkim@chosun.ac.kr (H.-L.K.); jhchung@chosun.ac.kr (J.-H.C.); 2 Department of Obstetrics and Gynecology, Chung-Ang University Gwang-myung Hospital, Chung-Ang University College of Medicine, Gwangmyeong-si 14353, Republic of Korea; shygirl1227@naver.com; 3 Department of Physiology, Dong-A University College of Medicine, Busan 49201, Republic of Korea

**Keywords:** chronic kidney disease, neck circumference, obesity, Korean National Health and Nutrition Examination Survey

## Abstract

Chronic kidney disease (CKD) is a major public health problem and a leading cause of cardiovascular disease and death. Early recognition and management of CKD risk factors are necessary to prevent its onset and progression. Neck circumference (NC) is a non-invasive and easily accessible anthropometric measure associated with central obesity and subcutaneous fat accumulation in the upper body. Our study aimed to explore the relationship between NC and the prevalence of CKD using data from the nationally representative Korea National Health and Nutrition Examination Survey (2019–2021). We analyzed data from 10,219 subjects (age > 19 years, no missing values). CKD was defined as an estimated glomerular filtration rate (eGFR) <60 mL/min/1.73 m^2^. Logistic regression analysis was performed, which revealed a significant association between NC and CKD prevalence even after adjusting for confounding factors, both when NC was considered a continuous variable (OR [95% CI], 1.11 [1.03–1.19]) and in quartiles (Q1 as reference; Q2 OR [95% CI], 1.23 [0.91–1.67]; Q3 OR [95% CI], 1.59 [1.16–2.18]; Q4 OR [95% CI], 1.70 [1.16–2.50]). Our findings suggest that NC could be a simple and effective anthropometric measurement for identifying individuals at risk for CKD.

## 1. Introduction

Chronic kidney disease (CKD) is diagnosed based on the presence of kidney damage or a reduced glomerular filtration rate (GFR), as indicated by clinical markers such as serum creatinine or cystatin C [1,2,3]. The incidence of CKD has surged significantly, primarily due to escalating rates of conditions such as type 2 diabetes mellitus (T2DM), hypertension (HTN), and obesity [4,5]. CKD is closely related to cardiovascular diseases and is an important cause of morbidity and mortality worldwide [4,6]. Additionally, the economic burden associated with CKD has witnessed a dramatic increase worldwide [7,8]. Managing CKD and its associated complications incurs substantial costs, which notably escalate with the progression of CKD stages or the initiation of dialysis [7,9]. Albuminuria, a crucial factor contributing to kidney damage and the progression of CKD, is also associated with metabolic syndrome (MetS) [10,11,12]. The Kidney Disease: Improving Global Outcomes (KDIGO) guideline categorizes albuminuria into microalbuminuria (spot urine albumin-to-creatinine ratio; uA/Cr 30–300 mg/g) and macroalbuminuria (uA/Cr > 300 mg/g) based on the extent of albumin excretion [1]. The presence of albuminuria signifies a disruption of the kidney filtration barrier and endothelial dysfunction, playing a significant role in the development of atherosclerosis and vascular lesions [11,13,14,15]. Hence, reducing the amount of albuminuria stands as a primary therapeutic target for patients with kidney diseases, such as diabetic kidney disease (DKD), non-diabetic CKD, and IgA nephropathy (IgAN) [16,17,18]. Therapeutically, the inhibition of the renin–angiotensin–aldosterone system (RAAS) using angiotensin-converting enzyme inhibitors (ACEis) or angiotensin receptor blockers (ARB) has shown effectiveness in reducing albuminuria and slowing the progression of kidney diseases [18,19,20]. Recently, emerging evidence showed that antidiabetic agents, such as sodium-glucose co-transporter-2 (SGLT2) inhibitors like empagliflozin and dapagliflozin, have demonstrated the ability to reduce the amount of albuminuria and inhibited CKD progression in patients with DKD and non-diabetic CKD [21,22]. Finerenone, a non-steroidal mineralocorticoid receptor antagonist, has also exhibited promising results in inhibiting the progression of CKD and reducing cardiovascular events in patients with T2DM, as evidenced by the FIDELIO-DKD trial [23]. However, despite these efforts, the prevalence of CKD and end-stage renal disease (ESRD) is rapidly increasing worldwide, including in Korea [5,24]. Importantly, a substantial portion of the population remains unaware of CKD [25], potentially delaying diagnosis and treatment, thereby exacerbating the disease’s progression. CKD, often asymptomatic until reaching an advanced stage [26], highlights the critical role of early detection in preventing progression and associated complications. Hence, there is a need for an easily accessible and non-invasive screening tool to facilitate the early identification of CKD prevalence.

Obesity is characterized by an excessive accumulation of fat in the body, which increases the risk of several diseases such as T2DM, HTN, dyslipidemia, and cardiovascular diseases [27,28,29]. In addition, it stands as an independent contributor to CKD advancement and is linked with albuminuria [30,31]. Assessing obesity incorporates diverse methods, from considering weight, waist/height ratio, waist circumference (WC), or body mass index (BMI) to evaluating visceral adipose tissue (VAT) [32,33,34]. The distribution of body fat serves as a more reliable indicator of metabolic and obesity-related complications compared to the total amount of body fat [35,36]. To gain a more accurate assessment of an individual’s overall health, additional measures such as body fat percentage, WC, or other health indicators should be considered. Previous studies using BMI as a marker of obesity have shown a significant association between BMI and the risk of ESRD independent of other risk factors such as age, sex, and smoking [30,37]. Individuals with a BMI below 30 may have a higher body fat percentage than people with a BMI of 30 or more [38]. Although BMI is the most commonly used parameter for assessing obesity, it has the following limitations: it does not reflect body fat distribution and cannot be applied to muscle mass. WC provides information about abdominal obesity and serves as one marker of MetS [39,40]. Despite its significance, WC has an inability to differentiate between subcutaneous and visceral fat deposition [41]. Visceral adipose tissue (VAT) can be validated through computed tomography (CT) and magnetic resonance imaging (MRI) scans, which are considered the most well-established imaging methods for quantifying abdominal fat [42]. While CT is effective in assessing VAT, its cost and exposure to radiation make it challenging to employ as a screening test.

Neck circumference (NC) is an easily accessible and widely available anthropometric measure that remains relatively consistent throughout the day [43,44]. Previous studies have shown that NC is associated with central obesity and upper body subcutaneous fat accumulation [45,46]. An increase in NC raised the risk of T2DM and cardiovascular diseases [47,48,49]. Furthermore, NC was associated with the development of CKD in overweight Korean individuals [50]. This study aimed to validate whether NC is related to the prevalence of CKD and to establish NC as an independent predictor of CKD in the general Korean population.

## 2. Materials and Methods

### 2.1. Study Population

**Figure 1:**
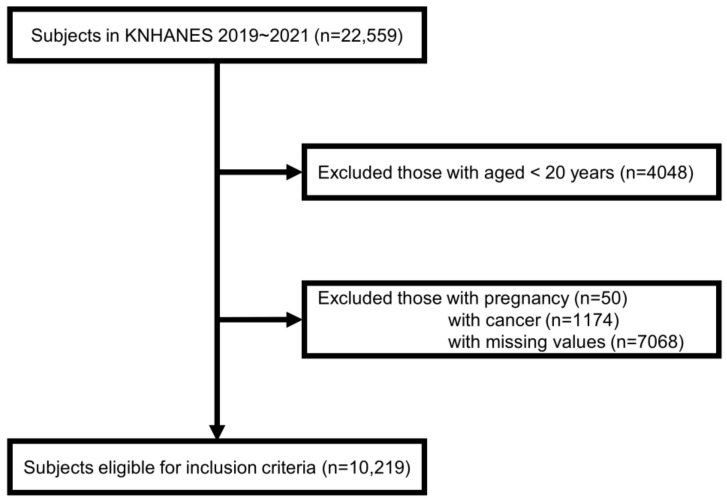
Flow diagram of the study subjects in this study.

This research analyzed data from the Korea National Health and Nutrition Examination Survey (KNHANES VIII), a comprehensive monitoring program overseen by the Korea Centers for Disease Control and Prevention (KCDC) [51,52]. The nationally representative cross-sectional KNHANES collected extensive data encompassing health status, behaviors, and dietary habits among the Korean populace. It serves as a fundamental resource for shaping health policies and acts as a robust research infrastructure, facilitating studies on risk factors and various diseases. We collected data from 22,559 participants in the KNHANES (2019–2021). Subjects were excluded based on the following criteria: participants under the age of 20 years (*n* = 4048), pregnant women (*n* = 50), those with cancer (*n* = 1174), and those with missing data (*n* = 7068). Consequently, this study analyzed a total of 10,219 subjects (Figure 1).

### 2.2. Demographic Characteristics and Lifestyle Survey

Information regarding population characteristics and health records, such as HTN, diabetes, and dyslipidemia, was gathered through questionnaires filled out by individuals themselves and through one-on-one interviews facilitated by trained personnel. Smoking status was categorized into current smokers and current non-smokers, including both ex-smokers and those who had never smoked. Subjects who consumed alcohol were categorized into two groups: non-alcohol drinkers, who had abstained from alcohol for a year or drank less than once a month, and alcohol drinkers who consumed alcohol more frequently than once a month. Exercise was categorized into two groups: regular exercise, defined as engaging in either ≥150 min of moderate-intensity exercise per week or ≥75 min of vigorous exercise per week, or a combination of exercises equivalent to these criteria (where 1 min of vigorous exercise equals 2 min of moderate-intensity exercise). Non-regular exercise included activity levels falling below the regular exercise criteria.

### 2.3. Anthropometric and Laboratory Measurements

To measure NC, all participants were seated in a chair with their hips, waist, and back against the backrest, head extended upward, and arms naturally at their sides. Next, NC was detected at once by palpating the Adam’s apple in males and the protruding thyroid cartilage in females. WC was checked at the midpoint between the bottom of the last rib and the top of the iliac crest on the right side of the mid-axillary line. Height and body weight were measured according to the KNHANES standard manual, and BMI was determined by dividing the body weight (kg) by the square of height (m^2^). Systolic and diastolic blood pressures (SBP and DBP, respectively) were measured three times by a trained nurse with the individual seated and their arm supported at heart level after 5 min of rest. Mean arterial pressure (MAP) was calculated as two-thirds of DBP plus one-third of SBP.

Blood samples were taken from participants following a minimum 8 h fasting period, while random spot urine samples were also collected. These samples underwent processing, refrigeration, and were promptly transported to the central laboratory within 24 h in cold storage. Hemoglobin levels were assessed using XN-9000 (Sysmex Corporation, Kobe, Japan). Serum concentrations of total cholesterol, high-density lipoprotein cholesterol (HDL-C), triglycerides (TG), and fasting glucose were measured using Labospect 008AS (Hitachi, Tokyo, Japan). Serum creatinine (sCr) concentration was tested using Cobas (Roche, Grenzach-Wyhlen, Germany). Urine albumin and creatinine levels were measured using LaboSpect 008AS (Hitachi, Japan).

### 2.4. The Definition of CKD and Metabolic Syndrome

CKD was defined and classified based on the Kidney Disease Improving KDIGO guidelines using estimated GFR (eGFR) [1]. eGFR was calculated using the Modification of Diet In Renal Disease (MDRD) formula. The following formula was used: eGFR (mL/min/1.73 m^2^) = 175 × sCr − 1.154 × age − 0.203 × 0.742 (if female) × 1.212 (if black). The GFR categories were divided into G1 (eGFR ≥ 90 mL/min/1.73 m^2^), G2 (eGFR 60–89 mL/min/1.73 m^2^), G3 (eGFR 30–59 mL/min/1.73 m^2^), G4 (eGFR 15–29 mL/min/1.73 m^2^), and G5 (eGFR < 15 mL/min/1.73 m^2^). Subjects with eGFR < 60 mL/min/1.73 m^2^ (G3, G4 and G5) were classified as patients with CKD. Diagnosis of MetS was based on the National Cholesterol Education Program-Adult Treatment Panel III (NCEP-ATP III) MetS diagnostic criteria [39] and the diagnostic criteria for abdominal obesity in Koreans [53]. MetS was diagnosed in subjects who exhibited three or more of the following five risk factors: (1) elevated fasting glucose (≥100 mg/dL), (2) high BP (DBP ≥ 85 mmHg or SBP ≥ 130 mmHg), (3) hypertriglyceridemia (TG ≥ 150 mg/dL), (4) reduced HDL-C (<50 mg/dL for women and <40 mg/dL for men), and (5) abdominal obesity (WC ≥ 85 cm for women and ≥90 cm for men).

### 2.5. Statistical Analysis

Continuous variables are reported as mean ± standard deviation, and categorical variables are presented as number of cases with percentage. We conducted a comparison of baseline characteristics among the NC quartiles by employing one-way analysis of variance (ANOVA) for continuous variables and chi-square tests for categorical variables. Univariable and multivariable logistic regression analyses were performed to investigate the association between NC and CKD status. The findings are displayed in terms of odds ratios (OR) along with their corresponding 95% confidence intervals (CI). Multivariable analysis included two models with progressively adjusted confounding factors. Model 1 was adjusted for sex, age, uA/Cr, BMI, WC, current smoking status, alcohol consumption, regular exercise, hypertension, diabetes, and dyslipidemia. Model 2 was adjusted for sex, age, uA/Cr, BMI, WC, current smoking status, alcohol consumption, regular exercise, MAP, glucose, and total cholesterol. All analyses were conducted using the SPSS software version 20 (IBM Corporation, Armonk, NY, USA), and graphs were drawn using R 4.3.0 (https://www.r-project.org, accessed on 4 November 2023, The R Foundation for Statistical Computing, Vienna, Austria). Statistical significance was set at *p* < 0.05.

## 3. Results

### 3.1. Characteristics of Study Participants

A total of 10,219 patients (average age of 59.7 ± 11.6 years and 55.7% were female) were included in this study. The participants were divided into quartiles based on NC and labeled as quartiles 1, 2, 3, and 4. The mean NC for each group was 32.60 cm, 34.42 cm, 35.63 cm, and 37.98 cm, respectively. The baseline characteristics of the study population according to the NC quartiles are shown in Table 1. Quartile 4 represents the youngest group, while quartile 2 corresponds to the oldest among the four groups, with mean ages of 58.68 and 60.02 years, respectively. Subjects with a higher NC tended to exhibit HTN, diabetes, dyslipidemia, and MetS, along with a higher BMI and WC (Table 1). Additionally, glucose, triglyceride, uA/Cr, and hemoglobin levels tended to increase, whereas HDL-C levels decreased in participants with a larger NC (Table 1).

### 3.2. Association between NC and Prevalence of CKD

**Figure 2:**
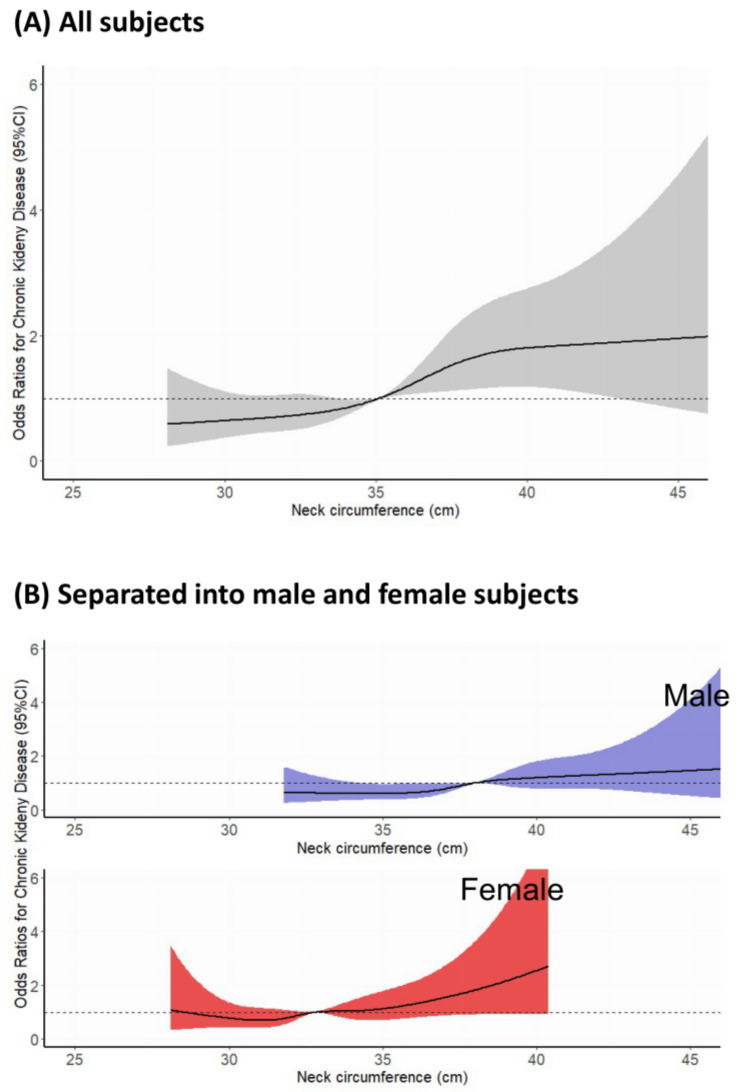
Restricted cubic spline for association between neck circumference and chronic kidney disease. (**A**) All subjects; (**B**) separated into male and female subjects. The dot lines mean reference value (odds ratio = 1). The color blue denotes male, while red signifies female.

Restricted cubic spline analysis revealed a correlation between a larger NC and the prevalence of CKD in all participants (Figure 2A) as well as in subgroup analyses for both males and females (Figure 2B). To demonstrate the association between NC and CKD prevalence, logistic regression analysis was performed. In univariable logistic regression, NC exhibited a significant association with the prevalence of CKD both as a continuous value (OR = 1.07, 95% CI [1.05–1.10], *p* < 0.001) and quartiles, except for the second quartile (Q1 as a reference; Q2: OR = 1.19, 95% CI [0.91–1.55], *p* = 0.20; Q3: OR = 1.47 95% CI [1.14–1.89], *p* = 0.003; Q4: OR = 1.43, 95% CI [1.10–1.84], *p* = 0.006) (Table 2). In multivariable logistic analysis, NC was a significant independent risk factor for the prevalence of CKD in both Model 1 (OR = 1.11, 95% CI [1.03–1.19], *p* = 0.004]) and Model 2 (OR = 1.14, 95% CI [1.06–1.22], *p* ≤ 0.001), based on continuous values (Table 2). The prevalence of CKD according to NC quartiles is shown in Table 2. The third and fourth quartiles were significantly associated with the risk of prevalence of CKD compared to the first quartile after adjusting, both in Model 1 (Q1 as a reference; Q2: OR = 1.23, 95% CI [0.91–1.67], *p* = 0.17; Q3: OR = 1.59 95% CI [1.16–2.18], *p* = 0.004; Q4: OR = 1.70, 95% CI [1.16–2.50, *p* = 0.007]) and Model 2 (Q1 as a reference; Q2: OR = 1.27, 95% CI [0.94–1.71], *p* = 0.12; Q3: OR = 1.68 95% CI [1.23–2.31], *p* = 0.001; Q4: OR = 1.85, 95% CI [1.26–2.71], *p* = 0.002). Consistent with univariate analysis, the second quartile did not show a significant association.

### 3.3. Correlation between Neck Circumference and Incidence of CKD, as Revealed by Subgroup Analysis

Subsequently, we conducted subgroup analyses based on sex, age, and MetS status (Table 3). Consistent with the restricted cubic spline curve (Figure 2B), subgroup analysis based on sex showed a significant correlation between NC and the prevalence of CKD (Table 1). Among participants > 65 years of age, there was a significant association between NC and the prevalence of CKD (Model 1, OR = 1.09, 95% CI [1.00–1.17], *p* = 0.04; Model 2, OR = 1.11, 95% CI [1.03–1.20] *p* = 0.01), while there was no association among those < 65 years (Model 1, OR = 1.01, 95% CI [0.87–1.17], *p* = 0.93; Model 2, OR = 1.04, 95% CI [0.89–1.20], *p* = 0.63) (Table 3). Among those with MetS, a significant relationship was observed between NC and the prevalence of CKD (Model 1, OR = 1.12, 95% CI [1.02–1.24], *p* = 0.02; Model 2, OR = 1.13, 95% CI [1.02–1.25], *p* = 0.02) (Table 3). However, among participants without MetS, a significant association between NC and the prevalence of CKD was found only in Model 2 (Model 1, OR = 1.10, 95% CI [1.00–1.22], *p* = 0.06; Model 2, OR = 1.13, 95% CI [1.02–1.25], *p* = 0.02) (Table 3).

## 4. Discussion

CKD is a notable risk factor for morbidity and mortality, notably elevating the risk of cardiovascular diseases compared to the general population [6,54]. Early recognition and management of CKD risk factors such as diabetes, HTN, and MetS play a pivotal role in preventing the onset and progression of CKD. The development of an easy and accurate screening tool that can be applied to the general population is crucial for effective management.

In this large-scale nationwide cross-sectional study, we showed a sustained, significant correlation between NC and CKD prevalence, even after adjusting for potential confounding factors such as sex, age, uA/Cr, BMI, WC, hypertension, diabetes, and dyslipidemia. Additionally, we explored this association by examining both continuous values and the four quartiles of NC. Numerous studies have consistently shown albuminuria is a key indicator of CKD prevalence [55,56]. Our multivariable logistic regression analysis revealed a significant association between NC and CKD prevalence, even after adjusting for uA/Cr as a confounding factor (Table 2 and Table 3). These findings reinforce the notion that NC is a potent predictor of CKD prevalence.

Previous studies have shown that obesity, measured using BMI, WC, and body fat mass, serves as an individual indicator of eGFR decline even after adjusting for other risk factors such as age, sex, race, and smoking [57,58]. NC is an anthropometric index for upper-body subcutaneous adiposity and central obesity [45,59]. Recent studies have suggested that NC is associated with the development and progression of CKD [60,61]. One study showed that NC can be used as a predictor of metabolic disorders and renal diseases [62]. Another study conducted in the Chinese population revealed a positive correlation between NC and eGFR decline [61]. The precise mechanism underlying the association between NC and eGFR decline remains to be fully validated; however, it has been suggested that free fatty acids (FFAs) may play a role. Upper-body subcutaneous adipose tissue releases a significantly greater amount of systemic FFAs than lower-body and visceral adipose tissues [35,63]. Elevated FFA levels are linked to insulin resistance, non-alcoholic fatty liver disease (NAFLD), atherosclerosis, and inflammation [64,65,66]. Moreover, high concentrations of FFAs can induce renal injury by promoting tubulointerstitial inflammation and fibrosis via reabsorption into the proximal tubules of the kidney [67]. Saturated FFAs induce oxidative stress, which leads to mitochondrial dysfunction in podocytes, subsequent podocyte injury, and glomerular damage [68,69]. MetS is closely linked to heightened insulin resistance and is a well-established risk factor for the development and progression of CKD [70,71,72,73,74]. While previous studies have identified NC as a marker of MetS [75,76]; our results not only demonstrated a persistent association between NC and CKD even after adjusting for MetS (Table 2) but also uncovered a significant association between CKD prevalence and NC among subjects not diagnosed with MetS (Table 3). These findings suggest that NC could be a useful tool for early CKD detection and risk stratification.

Subgroup analysis of participants aged >65 years revealed an independent association between NC and prevalence of CKD (Table 3). These results may be attributed to the prolonged exposure of older individuals to high FFA levels, leading to extended periods of renal injury. Alternatively, this could be attributed to the increased vulnerability of older individuals to FFAs. Consequently, a larger NC in individuals aged > 65 years emphasizes the importance of controlling risk factors, including the management of hypertension, diabetes, and dyslipidemia.

The clinical application of NC has expanded in recent years to include the prediction of insulin resistance in postmenopausal women with polycystic ovarian syndrome, liver disease, and thyroid disease [77,78]. However, further research is needed to refine the application of NC and validate its underlying mechanism. This study had several limitations. First, this was a cross-sectional study, which implies that we cannot establish a causal relationship between NC and CKD incidence. Second, this study relied on self-reported information for several factors, such as smoking, alcohol consumption, and exercise habits, which might have introduced a certain degree of bias. Third, a diagnosis of CKD requires a sustained GFR below 60 (mL/min/1.73 m^2^) for a minimum of three months [1]. While the KNHANES predominantly examined community-dwelling populations with a low likelihood of acute kidney injury [79], this study measured creatinine only once. As a result, the number of CKD cases may have been overestimated. Nevertheless, this study had a large sample size and was adjusted for various confounding factors to explore the relationship between NC and the prevalence of CKD in Koreans. Contrary to these limitations, this study had several strengths. First, it had a large sample size and comprehensive adjustments for various confounding factors to investigate the relationship between NC and prevalence of CKD in the Korean population. This focus is particularly crucial as CKD symptoms often remain undetected until the disease has significantly progressed. Second, we demonstrated that NC is associated with CKD prevalence by analyzing it not only as a continuous variable, but also in quartiles. Interestingly, we found that the second quartile did not show a significant association with CKD prevalence compared to the first quartile. These results suggest the need for further studies to determine the cut-off values of NC for increasing CKD risk. Additionally, educating the public about NC as an indicator of obesity and a risk factor for CKD can improve the management of CKD risk factors, particularly obesity. This heightened awareness could effectively reduce the CKD prevalence and alleviate the financial burden associated with CKD.

## 5. Conclusions

This study robustly demonstrated a significant association between NC and CKD prevalence in the general Korean population, even after adjusting for potential confounding factors. Our findings underscore the clinical significance of NC as a simple, non-invasive, and cost-effective anthropometric measure for identifying individuals at increased risk of CKD. Considering its ease of measurement and strong association with CKD prevalence, NC could serve as a valuable screening tool for detecting CKD. Further studies are needed to determine the specific cut-off values for CKD risk in males and females.

## Figures and Tables

**Table 1:** Baseline characteristics of study population.

Study Population (*n* = 10,219)	Quartile 1 (*n* = 2718)	Quartile 2 (*n* = 2473)	Quartile 3 (*n* = 2505)	Quartile 4 (*n* = 2523)	*p*-Value
Sex (%)					0.684
Male	1195 (44.0%)	1112 (45.0%)	1088 (43.4%)	1128 (44.7%)	
Female	1523 (56.0%)	1361 (55.0%)	1417 56.6%)	1395 (55.3%)	
Age (years)	59.98 ± 11.98	60.02 ± 11.75	59.90 ± 11.49	58.68 ± 11.19	<0.001
Blood analysis					
Hemoglobin (g/dL)	13.58 ± 1.50	13.79 ± 1.54	13.85 ± 1.55	14.06 ± 1.54	<0.001
Glucose (mg/dL)	99.65 ± 19.53	102.95 ± 22.59	105.93 ± 24.70	112.04 ± 28.18	<0.001
Total cholesterol (mg/dL)	191.27 ± 38.12	192.11 ±39.91	193.04 ± 41.46	190.14 ± 41.88	0.07
HDL cholesterol (mg/dL)	55.43 ± 13.54	51.96 ± 12.60	50.47 ± 12.37	47.76 ± 10.97	<0.001
Triglyceride (mg/dL)	112.40 ± 82.80	126.88 ± 107.19	142.51 ± 116.62	163.89 ± 119.36	<0.001
eGFR	90.26 ± 18.65	88.24 ± 17.71	86.30 ± 20.09	86.82 ± 18.11	<0.001
Urine albumin-to-creatinine ratio (mg/g)	0.23 ± 1.37	0.24 ± 1.35	0.31 ± 1.68	0.34 ± 1.62	0.03
Body measurements					
Body mass index (kg/m^2^)	21.32 ± 2.16	23.36 ± 2.05	24.81 ± 2.15	27.80 ± 3.07	<0.001
Waist circumference (cm)	77.48 ± 7.56	83.42 ± 7.05	87.14 ± 6.95	94.73 ± 8.12	<0.001
Neck circumference (cm)	32.60 ± 2.47	34.42 ± 2.59	35.63 ± 2.68	37.98 ± 3.09	<0.001
Health interview (%)					
Current smoker	401 (14.8%)	331 (13.4%)	340 (13.6%)	338 (13.4%)	0.41
Alcohol drinking	1033 (38.0%)	952 (38.5%)	989 (39.5%)	1013 (40.2%)	0.39
Regular exercise	1035 (38.1%)	971 (39.3%)	1006 (40.2%)	936 (37.1%)	0.13
Underlying diseases (%)					
Hypertension	847 (31.2%)	955 (38.6%)	1116 (44.6%)	1411 (55.9%)	<0.001
Diabetes	325 (12.0%)	402 (16.3%)	515 (20.6%)	774 (30.7%)	<0.001
Dyslipidemia	1065 (39.2%)	1188 (48.0%)	1371 (54.7%)	1654 (65.6%)	<0.001
Metabolic syndrome	303 (11.1%)	592 (23.9%)	953 (38.0%)	1503 (59.6%)	<0.001
GFR categories (%)					<0.001
G1	1325 (48.7%)	1067 (43.1%)	992 (39.6%)	1020 (40.4%)	
G2	1283 (47.2%)	1288 (52.1%)	1367 (54.6%)	1360 (53.9%)	
G3	102 (3.8%)	113 (4.6%)	137 (5.5%)	135 (5.4%)	
G4	6 (0.2%)	4 (0.2%)	6 (0.2%)	7 (0.3%)	
G5	2 (0.1%)	1 (0.0%)	3 (0.1%)	1 (0.0%)	

Values for continuous variables presented as mean ± standard deviation. *p* values of sex, current smoker, alcohol drinking, regular exercise, hypertension, diabetes, dyslipidemia, metabolic syndrome, and chronic kidney disease calculated using chi-squared test. *p* values of hemoglobin, glucose, total cholesterol, triglyceride, eGFR, urine albumin-to-creatinine ratio, body mass index, waist circumference, and neck circumference calculated using one-way ANOVA.

**Table 2:** Association between neck circumference and chronic kidney disease.

Neck Circumference	Crude Model		Adjusted Model *			
			Model 1 ^a^		Model 2 ^b^	
	OR (95% CI)	*p*-Value	OR (95% CI)	*p*-Value	OR (95% CI)	*p*-Value
Quartiles of neck circumference						
Quartile 1	1 (reference)		1 (reference)		1 (reference)	
Quartile 2	1.19 (0.91–1.55)	0.20	1.23 (0.91–1.67)	0.17	1.27 (0.94–1.71)	0.12
Quartile 3	1.47 (1.14–1.89)	0.003	1.59 (1.16–2.18)	0.004	1.68 (1.23–2.31)	0.001
Quartile 4	1.43 (1.10–1.84)	0.006	1.70 (1.16–2.50)	0.007	1.85 (1.26–2.71)	0.002
Continuous values of neck circumference						
Neck circumference (cm)	1.07 (1.05–1.10)	<0.001	1.11 (1.03–1.19)	0.004	1.14 (1.06–1.22)	<0.001

* The adjusted model was split into two distinct models: Model 1 and Model 2. ^a^ Model was adjusted for sex, age, body mass index, waist circumference, alcohol drinking, current smoking status, regular exercise, urine albumin-to-creatinine ratio, hypertension, diabetes, and dyslipidemia. ^b^ Model was adjusted for sex, age, body mass index, waist circumference, alcohol drinking, current smoking status, regular exercise, urine albumin-to-creatinine ratio, mean arterial pressure, glucose, and total cholesterol.

**Table 3:** Subgroup analysis for association between neck circumference and chronic kidney disease.

Subgroup	Crude Model		Adjusted Model *				*p* for Interaction
			Model 1 ^a^		Model 2 ^b^		
	OR (95% CI)	*p*-Value	OR (95% CI)	*p*-Value	OR (95% CI)	*p*-Value	
Sex							0.45
Male (*n* = 4523)	0.99 (0.94–1.04)	0.78	1.11 (1.02–1.22)	0.02	1.14 (1.04–1.25)	0.01	
Female (*n* = 5696)	1.14 (1.07–1.22)	<0.001	1.12 (1.00–1.25)	0.05	1.15 (1.02–1.28)	0.014	
Age							0.57
Above 65 years (*n* = 3640)	1.09 (1.05–1.13)	<0.001	1.09 (1.00–1.17)	0.04	1.11 (1.03–1.20)	0.01	
Under 65 years (*n* = 6579)	1.16 (1.09–1.22)	<0.001	1.01 (0.87–1.17)	0.93	1.04 (0.89–1.20)	0.63	
Metabolic syndrome							0.05
Yes (*n* = 3351)	0.97 (0.94–1.01)	0.20	1.12 (1.02–1.24)	0.02	1.13 (1.02–1.25)	0.02	
No (*n* = 6868)	1.11 (1.07–1.16)	<0.001	1.10 (1.00–1.22)	0.06	1.13 (1.02–1.25)	0.02	

* The adjusted model was split into two distinct models: Model 1 and Model 2. ^a^ Model was adjusted for sex, age, body mass index, waist circumference, alcohol drinking, current smoking status, regular exercise, urine albumin-to-creatinine ratio, hypertension, diabetes, and dyslipidemia. ^b^ Model was adjusted for sex, age, body mass index, waist circumference, alcohol drinking, current smoking status, regular exercise, urine albumin-to-creatinine ratio, mean arterial pressure, glucose, and total cholesterol.

## Data Availability

Information is accessible from the Korea National Health and Nutrition Examination Survey (KNHANES), organized by the Korea Centers for Disease Control and Prevention (KCDCP) and can be freely obtained from the KCDCP website (https://knhanes.cdc.go.kr, accessed on 4 November 2023).
